# Diagnosis and management of cancer therapy-related myocarditis in a young female: A case report and review of literature

**DOI:** 10.1186/s12872-024-03960-6

**Published:** 2024-06-10

**Authors:** Amir hossein Emami, Azin Alizadehasl, Masoud Sayad, Farnaz Shavandi, Parisa Firoozbakhsh, Shahla Meshgi, Kamran Roudini, Negar Dokhani

**Affiliations:** 1 Hematology-Oncology and Stem Cell Transplantation Research Center, Tehran University of Medical Sciences, Tehran, Iran; 2 Cardio-Oncology Research Center, Rajaie Cardiovascular Medical and Research Center, Iran University of Medical Sciences, Tehran, Iran; 3 Student Research Committee, Hamadan University of Medical Sciences, School of Medicine, Hamadan, Iran; 4 Cardiac Primary Prevention Research Center, Cardiovascular Diseases Research Institute, Tehran University of Medical Sciences, Tehran, Iran; 5 Rajaie Cardiovascular Medical and Research Center, Iran University of Medical Sciences, Tehran, Iran; 6 Department of internal medicine, Hematology and Medical oncology ward, Cancer research center, Imam Khomeini hospital complex, Tehran University of medical sciences, cancer institute, Tehran, Iran

**Keywords:** Cardiotoxicity, Sarcoma, Ewing, Myocarditis, Anthracyclines, Chemotherapy

## Abstract

**Background:**

The treatment of choice for Extra-osseous Ewing’s sarcoma/primitive neuroectodermal tumor (ES/PNET), a rare neoplasm, is the VAC/IE regimen. This regimen includes Doxorubicin, Vincristine, Cyclophosphamide, Ifosfamide, and Etoposide, all of which have cardiotoxic effects. Myocarditis, a potentially threatening side effect following cancer therapy, can be accurately managed and diagnosed.

**Case Presentation:**

In the current study, we report the case of a 19-year-old female with a mass on the abdominal wall, diagnosed with ES/PNET. She was treated with the VAC/IE regimen. A month after the last session of chemotherapy, she experienced dyspnea. Upon evaluation, a high level of troponin and a low left ventricular ejection fraction (LVEF) were detected via transthoracic echocardiography. She was treated with anti-heart failure drugs, but the response was unsatisfactory. The possibility of Cancer therapy-related myocarditis was suspected, and cardiac magnetic resonance imaging (CMR) confirmed acute myocarditis. This patient exhibited a significant response to intravenous immunoglobulin (IVIG), with her LVEF improving from 30–35% to 50% within three months.

**Conclusion:**

In this case, based on negative tests and the absence of viral signs and symptoms, Cancer therapy-related myocarditis is highly suspected as the cause of myocarditis. This case underscores the importance of accurately utilizing CMR as a non-invasive method for diagnosing myocarditis. It effectively highlights the identification of reversible myocarditis with appropriate treatment and the notable response to IVIG, suggesting its potential as a favorable treatment for myocarditis in younger patients.

**Supplementary Information:**

The online version contains supplementary material available at 10.1186/s12872-024-03960-6.

## Background

Cancer therapy-related cardiotoxicity is a notable adverse effect of chemotherapy and radiotherapy, associated with high morbidity and mortality rates [1]. Familiarity with these cardiac effects and their management is crucial for preserving the health of cancer patients.

## Case Presentation

A 19-year-old female presented to the hospital with a complaint of dyspnea categorized as mMRC grade III. This dyspnea initially began one month ago at mMRC grade I but has worsened over the last week. At presentation, her pulse rate was 110 beats per minute, blood pressure measured at 90/70 mmHg, and her oxygen saturation was 90% on room air. Additionally, she exhibited tachypnea. During the cardiovascular examination, S3 sound was auscultated on the apex, and crackles were noted during lung auscultation.

Last year, she noticed a small, painless, and non-mobile mass on her left lower abdominal wall that initially resembled a lipoma but gradually increased in size over two months, prompting further evaluation. Following surgery in July 2022, her biopsy showed a malignant tumor composed of atypical round cells with positive CD99 and positive Vimentin cytoplasmic reactions, while Desmin, LCA, WT-1, and Myogenin tests returned negative results.

she was diagnosed with Extra-osseous Ewing’s sarcoma/primitive neuroectodermal tumor (ES/PNET). Subsequently, she underwent chemotherapy, which included (Vincristine (2 mg/m^2^$\sim$3.5 mg D1), Doxorubicin (37.5 mg/m^2^$\sim 60 \text{m}\text{g}$D1-D2, totally: 600 mg), and Cyclophosphamide (1200 mg/m^2^$\sim2000\text{m}\text{g}$ D1)) in weeks 1-5-9-15-17, alternating with Ifosfamide (1800 mg/m^2^$\sim3000\text{m}\text{g}$ D1-D5) and Etoposide (100 mg/m^2^$\sim150\text{m}\text{g}$ D1-D5). This treatment regimen began in the following month after her diagnosis. The onset of dyspnea occurred one month after the last dose of chemotherapy. She underwent periodic echocardiography during the seven sessions of chemotherapy, and her last echocardiogram, performed before the final chemotherapy session, revealed normal LV systolic and diastolic function (EF: 55–60%). This procedure was not conducted at our center, and we only have access to the reports, as the videos are unavailable.

Her ECG showed sinus tachycardia without significant ST-T changes. Tissue doppler imaging at this centre revealed severely reduced LV systolic function (LVEF: 30%), LVEDVi: 53 ml/m2, LVESV: 70 ml, LVEDV: 78 ml, mild LV diastolic function (septal e’: 5.2 cm/s, lateral e’: 15 cm/s, LA volume: 43 ml/m2), global hypokinesia, moderate to severe RV systolic dysfunction (RVsm: 9 cm/s, TAPSE: 12 mm), and severe MR. (Supplementary file, Video-1: Short axis view of LV in the level of papillary muscle, EF: 30–35%, Video-2: Four chambers view, moderate to severe MR. Video-3: Four chambers view, global hypokinesia) With the probability of cancer therapy-related cardiomyopathy, standard anti-heart failure treatment was initiated based on GDMT (guideline-directed medical therapy). This treatment consisted of Spironolactone (25 mg daily), Empagliflozin (5 mg daily), Sacubitril-Valsartan (24/26 mg ½ tab twice a day), and Lasix (100 mg infusion in 24 h). Additionally, her troponin level was reported as 130 ng/L out of the hospital, and our centre’s HS-troponin level reported 0.44 µg/L (positive > 0.16).

Given this elevated troponin level and the absence of the expected response to the anti-HF treatment, a cardiac MRI was performed to investigate the possibility of myocarditis.

The patient had no recent history of viral infections, exhibited no current signs and symptoms of one, and the probability of viral myocarditis was very low based on the laboratory tests and clinical symptoms. Additionally, her viral, bacterial, and rheumatologic tests were negative (Table 1). Her vaccinations was in accordance with the country’s vaccination plan, and she had not receiveed any vaccine since the last dose of the influenza vaccine last year. She also denied the use of herbal supplements or any other treatments outside of the standard.

**Table 1:** Laboratory tests

Test	Result	Normal range
Influenza A/B PCR	Negative	
Covid-19 PCR ☓2	Negative	
HIV-Ab	Non-reactive	
HBSAg		
HCV-Ab		
TSH	0.53 µIU/ml	0.35–4.99 µIU/ml
T3-total	0.65 ng/ml	0.35–1.93 ng/ml
T4- total	8.69 µg/dl	4.87–11.72 µg/dl
B/C ☓3	Negative	
U/C	Negative	
CBC	WBC:7800 cells/ µL	4500–11000 cells/ µL
	Neut:87%	
	Lym:3%	
	Hb:13.9 g/dL	12-15.6 g/dL
	Plt:151000/ mm^3^	150000–450000
NT-proBNP	9688 pg/ml	under 75 years< 125 pg/ml
CRP	8 mg/L	< 6 mg/L
CPK-MB	6.5 IU/L	< 24 IU/L
Ds-DNA	Negative	
ANA		
RF		
Anti CCP		
P-ANCA		
C-ANCA		
ESR	11 mm/h	Female under 50 years < 30

**Fig. 1:**
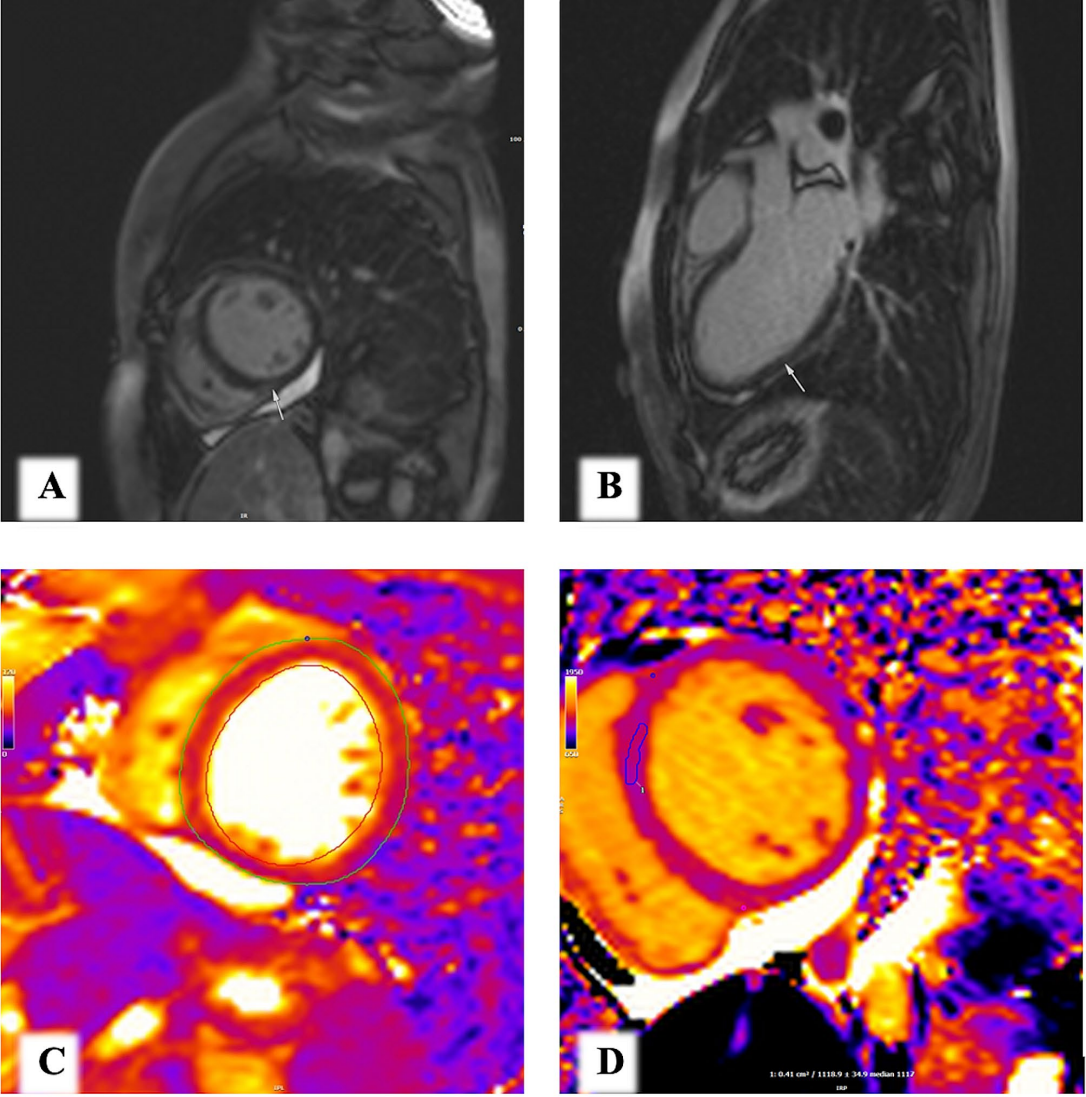
Cardiac magnetic resonance

The CMR revealed active myocarditis (Fig. 1) (A&B) Short axis at the level of mid LV and 3-chamber view of LGE sequence respectively show subepicardial to midmyocardial gadolinium enhancement of inferior and inferolateral LV wall (as denoted by marker) in favor of myocardial inflammation. (C&D) T2 &T1 weighted map sequences respectively showing significant diffuse elevated T1 and T2 values, global T2 excluding the blood pool was 56 milliseconds (ref:50ms) and T1 at the ROI was 1108ms (ref:1050ms). All the above findings indicate significant diffuse myocardial inflammation.(Siemens Magnetom sola 1.5T)). A treatment plan was initiated, which included Prednisolone at a dose of 1 mg/kg, as well as four sessions of Intravenous Immunoglobulin (IVIG) at 5 mg every day. Following the second session of IVIG, the patient’s condition began to improve, and her symptoms gradually subsided. After completing the four sessions of IVIG, the patient’s left ventricular ejection fraction (LVEF) had increased to 40%, then 45% One month later. In the last echocardiography, it was reported as Normal LV size with mild LV systolic dysfunction (LVEF:45–50%, LVESV: 62 ml, LVEDV: 82 ml), normal RV size and normal RV systolic function (RVsm:10 cm/s, TAPSE:19 mm) mild MR, septal e’: 10 cm/s, lateral e’: 15 cm/s, GLS;-14.6, LA volume: 22 ml. (supplementary file, Video-4: Short Axis view, EF: 45–50%., Video-5: Four Chambers view, Mild MR, Video-6: Four chambers view) She is currently receiving Sacubitril-Valsartan (24/26 mg half a tablet twice a day), empagliflozin 12.5 mg daily, carvedilol 3.125 mg twice daily, and prednisolone 15 mg daily.

## Discussion and conclusion

In this article, we present a case of a rare neoplasm with an uncommon therapy side effect, along with its timely management and treatment. Extra-osseous Ewing’s sarcoma/primitive neuroectodermal tumor (ES/PNET), which belongs to the Ewing sarcoma family of tumors (ESFTs), is a rare small round cell carcinoma. PNETs are most common in children and adolescents, with no significant gender predisposition. The incidence rates range from 0.15 per 100,000 in those younger than 5 years old, decreasing to 0.03 per 100,000 in young adolescents up to 19 years old [2–4].

The first-line treatment regimen for Ewing sarcoma is VAC/IE [5], which includes Vincristine, Doxorubicin, Cyclophosphamide, Ifosfamide, and Etoposide.

The combination of negative viral, bacterial, and rheumatologic tests, along with the absence of a history of herbal drugs, substances, and recent vaccinations, leads us to suspect cancer therapy-related myocarditis, especially considering the patient’s previous exposure to these drugs.

Vinca alkaloids, such as vincristine, are microtubule destabilizing agents, mostly prescribed in hematologic malignancies, brain neoplasms, and solid tumors [6, 7]. Their cardiotoxic effects mainly present as myocardial ischemia and infarction, occurring during or shortly after treatment, and are mainly attributed to cellular hypoxia and subsequent coronary artery vasospasm caused by the drug [8].

Doxorubicin, an anthracycline commonly used and highly effective for treating hematological malignancies and solid tumors, including Ewing sarcoma, can lead to cardiotoxicity. The cardiotoxicity is primarily dose-dependent but may also occur early during treatment. Anthracycline-induced cardiotoxicity primarily results from topoisomerase-II inhibition and oxidative stress induced by reactive oxygen species. cardiotoxicity manifests in three distinct forms: immediate myo-pericarditis, occurring within the initial month of treatment or following a single dose which is rare; early-onset chronic progressive congestive heart failure (CHF); and late-onset cardiotoxicity, emerging several years after treatment. While the cardiotoxic effects of anthracyclines are well-known, myocarditis is considered a rare manifestation [9–13].

Cyclophosphamide, a nitrogen mustard alkylating agent with potent anti-neoplastic, immunosuppressive, and immunomodulatory properties, can cause a spectrum of cardiotoxic effects, mostly manifesting as tachyarrhythmias, hypotension, heart failure, myocarditis, and pericardial disease, typically presenting within 2–10 days of drug administration. These cardiotoxic effects mostly occur due to increased oxidative stress and direct endothelial injury caused by cyclophosphamide metabolites, leading to extravasation of plasma proteins, erythrocytes, and toxic metabolites [14–16].

Ifosfamide is another alkylating agent that presents its cardiac side effects mainly as heart failure and arrhythmia [17].

Etoposide’s cardiotoxic effects mainly presents as hypotension, or less frequently as myocardial ischemia and MI. Concurrent chemotherapy with other agents or a previous history of chemotherapy or mediastinal irradiation are known risk factors that predispose patients to etoposide-induced MI [18, 19].

Doxorubicin and Cyclophosphamide are the drugs reported to cause myocarditis as a cardiotoxic effect, each acting through different pathways and highly suspected as the cause of myocarditis in this case.

Myocarditis exhibits a range of clinical presentations, from asymptomatic cases to those posing a life-threatening condition [20]. Based on the 2022 ESC Guidelines on cardio-oncology, the diagnosis of myocarditis involves a new or significant elevation of cTn plus 1 major criterion or 2 minor criteria. In this case, the patient’s CMR was diagnostic for myocarditis based on the updated Lake Louise criteria, serving as a major criterion. Additionally, the patient exhibited the clinical syndrome of myocarditis and a decline in LV systolic function, fulfilling 2 minor criteria. Consequently, the cTn elevation with 1 major and 2 minor criteria led to the clinical diagnosis of Myocarditis, and the patient is in the recovery phase. This patient experienced symptomatic severe CTRCD, requiring hospitalization [21, 22].

The treatment for myocarditis typically involves diuretics, vasodilators, and remodeling therapy, such as ACE inhibitors or angiotensin II receptor blockers, beta-blockers, and aldosterone antagonists. Regular follow-up using echocardiography and cardiac magnetic resonance imaging is also essential. In this patient,  all these treatments were administered. However, the absence of a positive response led us to consider intravenous immunoglobulin (IVIG), which has shown favorable results in pediatric cases [23] Additionally, a meta-analysis by Huang et al. demonstrated that IVIG therapy improves in-hospital survival and left ventricular function recovery in acute myocarditis patients. It also enhances survival rates in those with acute fulminant myocarditis, providing further support for its efficacy [24].

### Electronic supplementary material

Below is the link to the electronic supplementary material.

Supplementary Material 1

Supplementary Material 2

Supplementary Material 3

Supplementary Material 4

Supplementary Material 5

Supplementary Material 6

## Data Availability

Data is provided within the manuscript or supplementary information files.

## Abbreviations

mMRC scale: Modified Medical Research Council scale
LGE: Late gadolinium enhancement
